# Editorial: Molecular Tracing of Aquatic Viruses: Where Epidemiology Needs to Meet Genomics

**DOI:** 10.3389/fmicb.2017.01498

**Published:** 2017-08-08

**Authors:** Jean-Christophe Avarre

**Affiliations:** ISEM, IRD, CNRS, Univ. Montpellier, EPHE Montpellier, France

**Keywords:** aquaculture, virus, genomics, tracing, epidemiology

Viruses are highly abundant in aquatic environments (Bergh et al., 1989; Suttle, 2007) and can infect a wide range of organisms, from plankton cells to whales (Suttle, 2005). If aquatic viruses can cause mortalities among wild fish and shellfish populations, the rapid (and often uncontrolled) development of intensive aquaculture over the last decades has been a major driver of the emergence of many viral diseases (Walker and Winton, 2010). As a result, viral diseases now constitute a real threat for the sustainability of this ever-growing worldwide industry. In addition to their devastating economic and social impact resulting in losses of several $US billion (Walker and Mohan, 2009), viruses may also have substantial environmental impacts on the surrounding ecosystems, either directly (Bunce and Norman, 2000; Dann et al., 2000) or indirectly (Le et al., 2005; Ali, 2006; Phan et al., 2011).

As stated by Walker and Winton, the extent of disease spread and impacts is greatly affected by the availability of suitable diagnostic tools for identification of the causative agent or by the ability to understand the genetic relation of the causative agent with other characterized pathogens (Walker and Winton, 2010). An example is the emergence of the white spot syndrome in 1992, which has spread to almost all shrimp-producing countries before its causative agent, the white spot syndrome virus, could be identified and fully characterized nearly 9 years later (Yang et al., 2001).

In this context, the objectives of this research topic were: (i) to illustrate how sequencing technologies and associated bioinformatics tools can be utilized to trace aquatic viruses, and (ii) to discuss the new opportunities they offer for understanding the emergence of viral diseases and controlling their spread.

The two first articles of this topic show how sequencing a single gene of a virus may help understand the ways it propagates between aquaculture settings. Sequence comparison of VP2 gene from infectious pancreatic necrosis virus isolates showed the existence of two clades that differed by a distinct signature motif in the hypervariable region, each motif being associated with a different level of infection (Mutoloki et al.). In the second paper, Abbadi et al. looked at the evolution of two viruses that cause high mortalities in rainbow trout farms, the infectious haematopoietic necrosis virus (IHNV) and the viral haemorrhagic septicaemia virus (VHSV). They analyzed the glycoprotein (G) gene sequences of many isolates collected over a period of nearly 30 years and concluded that the two viruses have distinct evolutionary rates. Integration of these molecular data with high-quality epidemiological information led to propose different patterns of virus spread among trout farms.

In a third article, Klafack et al. describe an improved method to genotype and trace the highly pathogenic Cyprinid herpesvirus 3. They developed a qPCR test that enables to discriminate between the Asian and European lineages, and found that this virus was able to switch between the Asian and European genotypes after many *in vitro* passages.

The following two articles discuss the ability of metagenomics to uncover the environmental factors that influence virus spread in aquatic environments and to identify novel pathogenic viruses. Most of our knowledge on the epidemiology of viral diseases in aquaculture so far is derived from studies carried out on infected aquatic organisms. Metagenomics offers the possibility to study the epidemiology of viral diseases outside their host species by the direct analysis of environmental samples, which in turn enables to follow the environmental factors that influence the composition of viral communities (a recent example is provided by Hwang et al. (2017). Understanding the factors (both natural and anthropogenic) that influence the epidemiology of viral diseases may ultimately lead to the design of rational disease control strategies, especially in the aquaculture context (Munang'andu). Likewise, metagenomics has greatly accelerated the pace of virus discovery in the recent years, and also appears valuable to design proactive diagnostic tools able to identify novel viruses before they cause disease outbreaks (Munang'andu et al.).

In the particular context of viral aquatic diseases, analysis of outbreaks requires typing methods that offer a high level of strain discrimination. As outlined in the sixth article, whole genome sequencing (WGS) represents the “ultimate” typing methodology in terms of discriminatory power (Bayliss et al.). WGS not only enables to resolve micro-evolutionary distances, but it also has the power to discover new and rare variations, including polymorphisms that arise during an outbreak or that evolve *in vivo* during an infection. It thus offers the possibility to track the emergence and spread of a given variant, while it may also provide predictive information concerning key phenotypic traits (Feil, 2015).

Finally, in the last article of this topic, Naville and Volff raise an interesting question that is often overlooked in the field of viral (re)emergence: the potential role of fish endogenous retroviruses. While several exogenous retroviruses have been clearly identified as etiological agents of some fish diseases (Lepa and Siwicki, 2011; Coffee et al., 2013), little is known about the association between endogenous retroviruses and diseases in fish, with the exceptions of the zebrafish endogenous retrovirus (Frazer et al., 2012) and the salmon leukemia virus that may have an endogenous origin (Eaton et al., 1994). Naville and Volff speculate that scrutiny of more fish genomes will certainly uncover new types of retroviruses, some of which with a potential for retained infectivity or reemergence, as was recently discovered in the human genome (Wildschutte et al., 2016).

To conclude, this research topic brings evidence that genomics has now become a routine tool for tracing viral pathogens. However, in spite of the promises brought by new sequencing technologies, it is critical to keep in mind that a molecular typing dataset, including whole genome sequencing, will rarely be fully informative if it is not associated with rich epidemiological (meta)data. Besides, both metagenomics and WGS are facing technological challenges when applied to aquatic viruses, which the third and fourth generations of sequencing will probably only partly overcome (Nkili-Meyong et al., 2016).

## Author contributions

The author confirms being the sole contributor of this work and approved it for publication.

### Conflict of interest statement

The author declares that the research was conducted in the absence of any commercial or financial relationships that could be construed as a potential conflict of interest.
